# Supplementary figures and images for: Litter inputs and standing stocks in riparian zones and streams under secondary forest and managed and abandoned cocoa agroforestry systems
Source: PeerJ. 2022 Dec 1;10:e13787. doi: 10.7717/peerj.13787 (PMC9744167; doi:10.7717/peerj.13787)

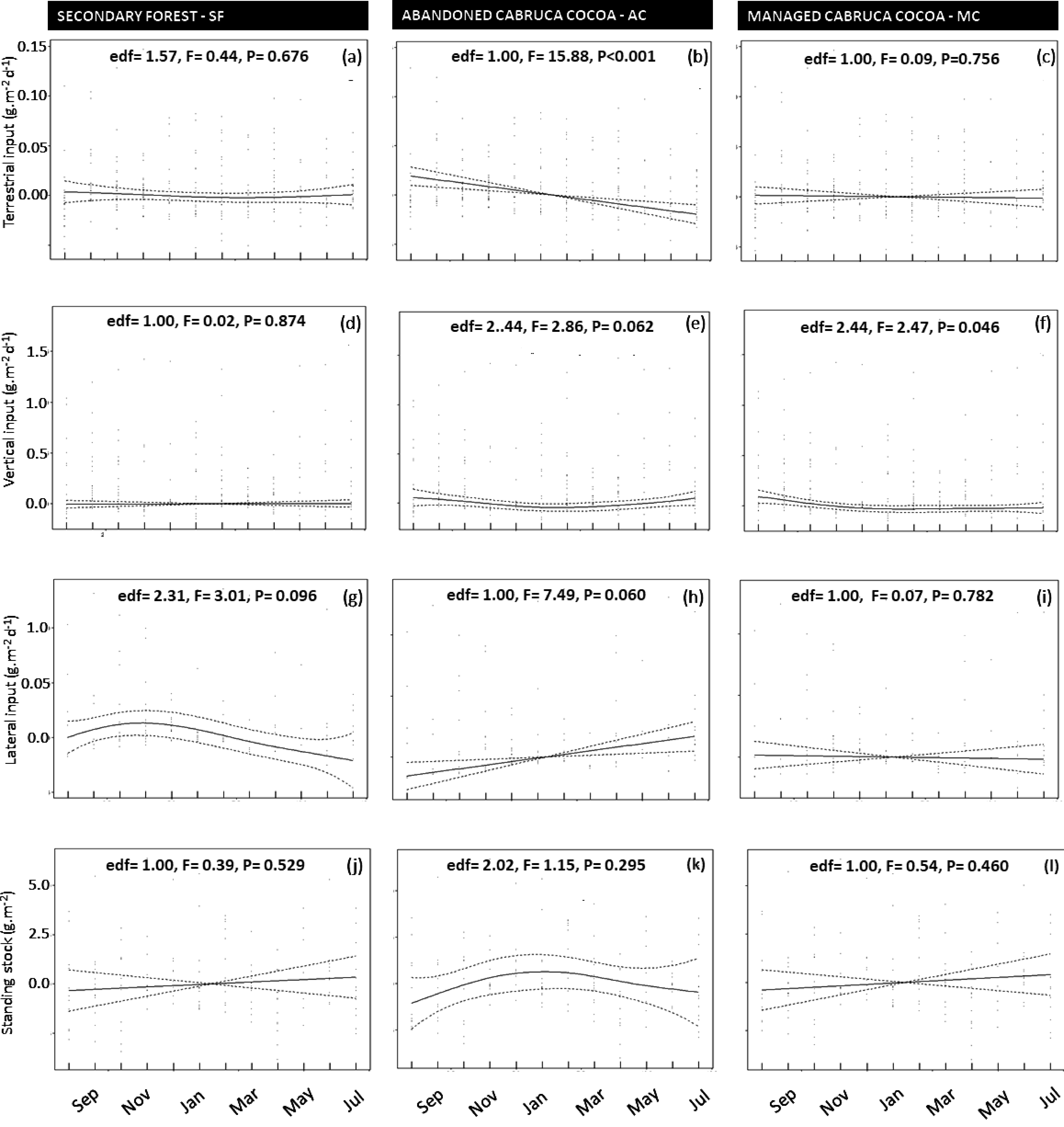

Supplement: Supplemental Information 1 — Also shown are F and P values as well as the effective degrees of freedom (edf) of GAMM analyses. Continuous lines are the GAMM smoothers and dotted lines indicate 95% confidence limits. [file peerj-10-13787-s001.jpg]

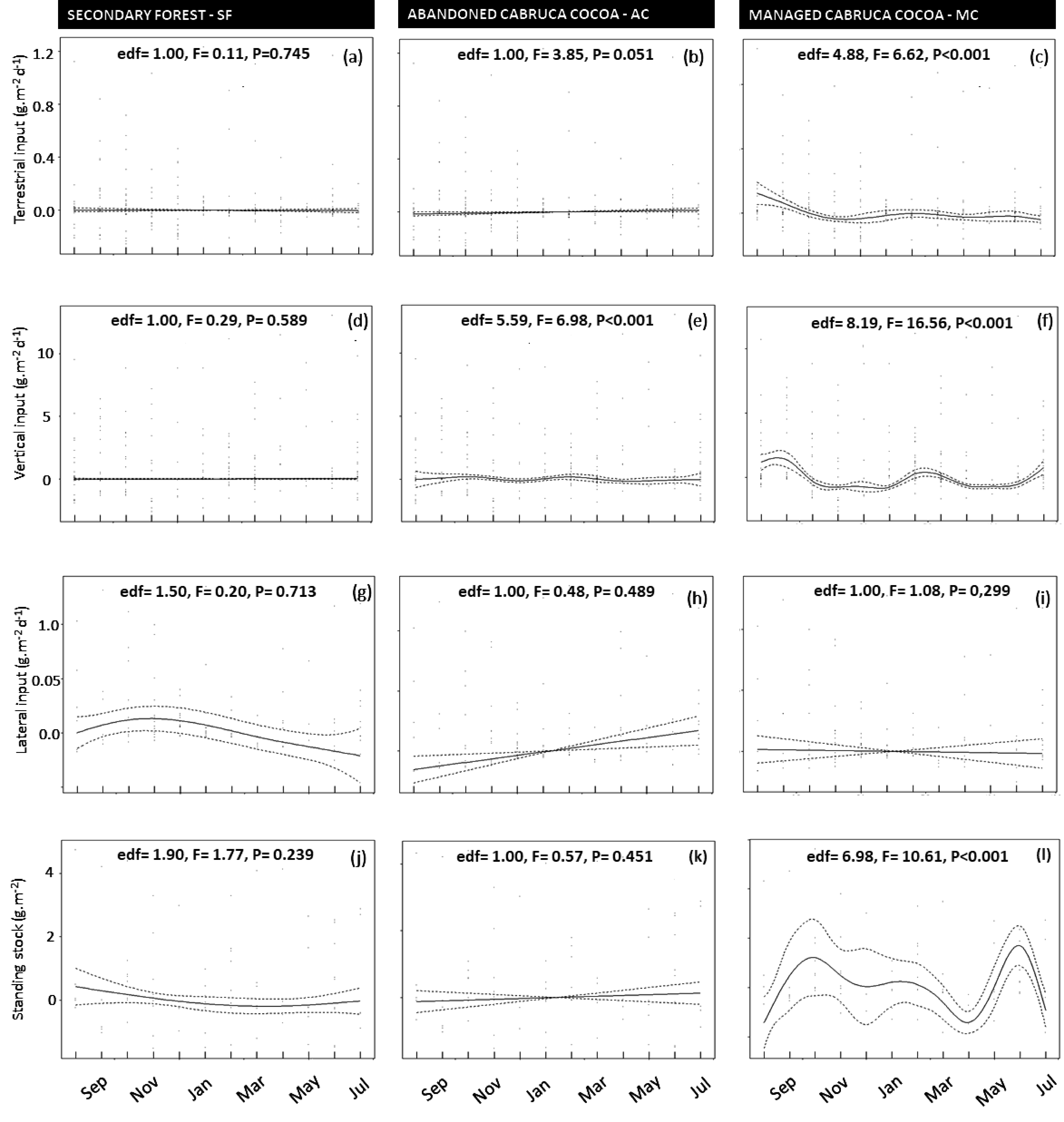

Supplement: Supplemental Information 2 — Also shown are F and P values as well as the effective degrees of freedom (edf) of GAMM analyses. Continuous lines are the GAMM smoothers and dotted lines indicate 95% confidence limits. [file peerj-10-13787-s002.jpg]

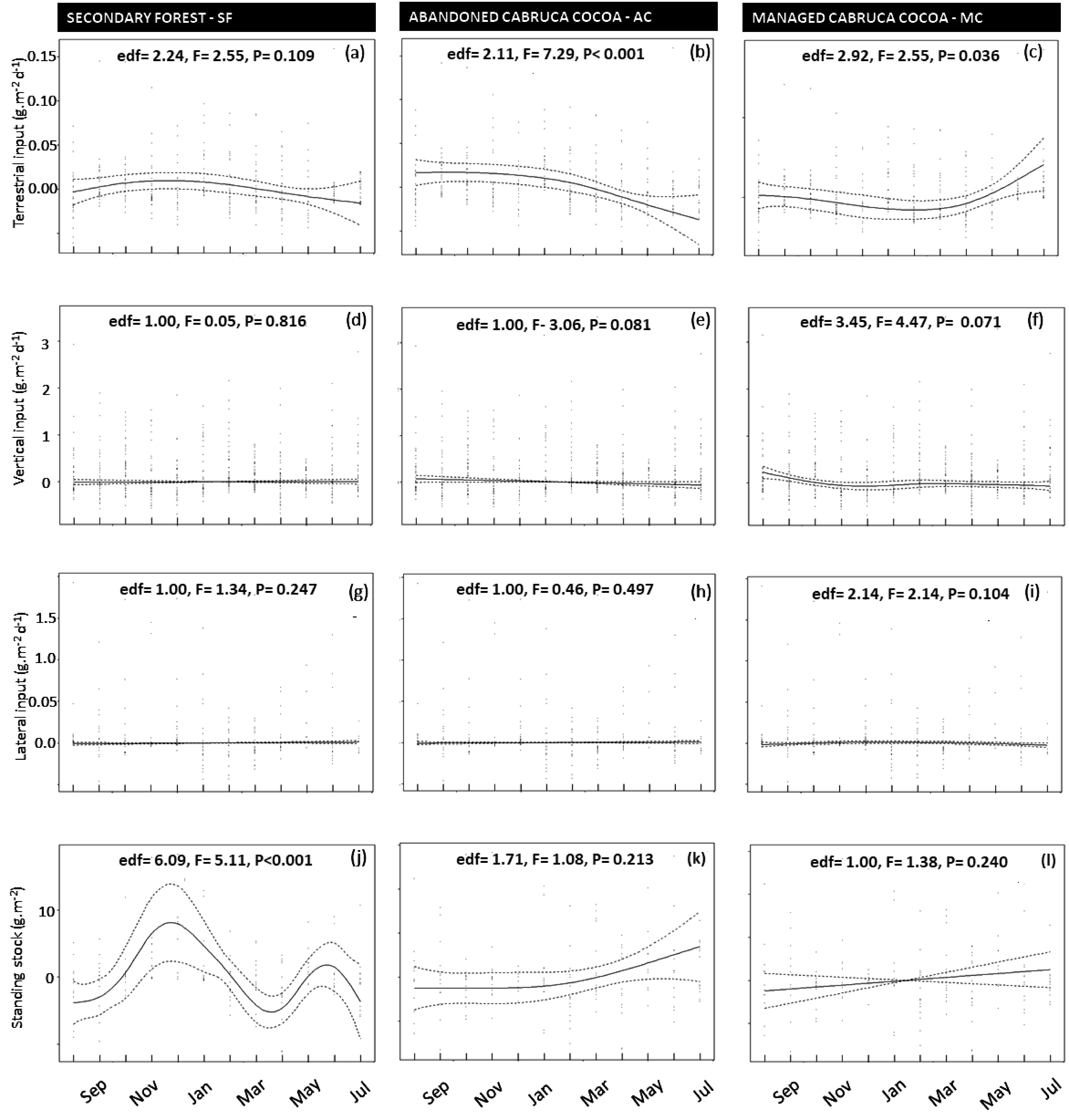

Supplement: Supplemental Information 3 — Also shown are F and P values as well as the effective degrees of freedom (edf) of GAMM analyses. Continuous lines are the GAMM smoothers and dotted lines indicate 95% confidence limits. [file peerj-10-13787-s003.jpg]
