# Supplementary material for: Litter inputs and standing stocks in riparian zones and streams under secondary forest and managed and abandoned cocoa agroforestry systems
Source: PeerJ. 2022 Dec 1;10:e13787. doi: 10.7717/peerj.13787 (PMC9744167; doi:10.7717/peerj.13787)
Supplement: Supplemental Information 5 — AIC = Akaike Information Criterion, BIC = Bayesian Information Criterion, logLik = log likelihood. [file peerj-10-13787-s005.pdf]

Table S1.

	Df	AIC	BIC	logLik	Deviatio	Chi Squared	Df	P(>Chi squared)
**A. Leaves**								
Null model	5	7478.7	7501.8	-3734.3	7468.7			
Time	8	7419.1	7456.1	-3701.6	7403.1	65.6	3	< 0.001
Null model	4	7481.7	7500.2	-3736.8	7473.7			
Site	8	7419.1	7456.1	-3701.6	7403.1	70.6	4	< 0.001
Null model	3	7533.4	7547.2	-3763.7	7527.4			
Site : Time	8	7419.1	7456.1	-3701.6	7403.1	124.3	5	< 0.001
**B. Branches**								
Null model	5	6155.8	6178.9	-3072.9	6145.8			
Time	8	6151.5	6188.5	-3067.8	6135.5	10.2	3	0.017
Null model	4	6150.1	6168.6	-3071.1	6142.1			
Site	8	6151.5	6188.5	-3067.8	6135.5	6.6	4	0.159
Null model	3	6155.2	6169.0	-3074.6	6149.2			
Site : Time	8	6151.5	6188.5	-3067.8	6135.5	13.6	5	0.018
**C. Reproductive**								
Null model	5	8083.8	8106.9	-4036.9	8073.8			
Time	8	8060.8	8097.8	-4022.4	8044.8	29.0	3	< 0.001
Null model	4	8146.2	8164.7	-4069.1	8138.2			
Site	8	8060.8	8097.8	-4022.4	8044.8	93.4	4	< 0.001
Null model	3	8151.5	8165.3	-4072.7	8145.5			
Site : Time	8	8060.8	8097.8	-4022.4	8044.8	100.6	5	< 0.001
**D. Miscellaneous**								
Null model	5	6153.0	6176.1	-3071.5	6143.0			
Time	8	6149.9	6186.8	-3066.9	6133.9	9.1	3	0.053
Null model	4	6155.1	6173.5	-3073.5	6147.1			
Site	8	6149.9	6186.8	-3066.9	6133.9	13.2	4	0.051
Null model	3	6157.5	6171.4	-3075.8	6151.5			
Site : Time	8	6149.9	6186.8	-3066.9	6133.9	17.6	5	0.055
