# Supplementary material for: Litter inputs and standing stocks in riparian zones and streams under secondary forest and managed and abandoned cocoa agroforestry systems
Source: PeerJ. 2022 Dec 1;10:e13787. doi: 10.7717/peerj.13787 (PMC9744167; doi:10.7717/peerj.13787)
Supplement: Supplemental Information 6 — AIC = Akaike Information Criterion, BIC = Bayesian Information Criterion, logLik = log likelihood [file peerj-10-13787-s006.pdf]

Table S2.

	Df	AIC	BIC	logLik	Deviation	Chi square	Df	P > (Chi square)
**A. Leaves**								
Null model	5	3485.5	3504.7	-1737.8	3475.5			
Time	8	3448.3	3478.9	-1716.1	3432.3	43.3	3	< 0.001
Null model	4	3465.2	3480.5	-1728.6	3457.2			
Site	8	3448.3	3478.9	-1716.1	3432.3	24.9	4	0.052
Null model	3	3494.4	3505.9	-1744.2	3488.4			
Site : Time	8	3448.3	3478.9	-1716.1	3432.3	56.1	5	< 0.001
**B. Branches**								
Null model	5	2613.9	2633	-1301.9	2603.9			
Time	8	2602.8	2633.4	-1293.4	2586.8	48.5	3	< 0.001
Null model	4	2643.3	2658.6	-1317.7	2635.3			
Site	8	2602.8	2633.4	-1293.4	2586.8	17.0	4	0.067
Null model	3	2649.8	2661.3	-1321.9	2643.8			
Site : Time	8	2602.8	2633.4	-1293.4	2586.8	57.9	5	< 0.001
**C. Reproductive**								
Null model	5	3405.8	3424.9	-1697.9	3395.8			
Time	8	3400.2	3430.8	-1692.1	3384.2	11.6	3	0.009
Null model	4	3423.1	3438.4	-1707.5	3415.1			
Site	8	3400.2	3430.8	-1692.1	3384.2	30.9	4	< 0.001
Null model	3	3424.1	3435.6	-1709.1	3418.1			
Site : Time	8	3400.2	3430.8	-1692.1	3384.2	34.0	5	< 0.001
**D. Miscellaneous**								
Null model	5	3099.2	3118.3	-1544.6	3089.2			
Time	8	3097.7	3128.3	-1540.9	3081.7	7.5	3	0.049
Null model	4	3099.3	3114.7	-1545.7	3091.3			
Site	8	3097.7	3128.3	-1540.9	3081.7	9.6	4	0.051
Null model	3	3097.9	3109.4	-1545.9	3091.9			
Site : Time	8	3097.7	3128.3	-1540.9	3081.7	10.1	5	0.047
