# Supplementary material for: Litter inputs and standing stocks in riparian zones and streams under secondary forest and managed and abandoned cocoa agroforestry systems
Source: PeerJ. 2022 Dec 1;10:e13787. doi: 10.7717/peerj.13787 (PMC9744167; doi:10.7717/peerj.13787)
Supplement: Supplemental Information 7 — AIC = Akaike Information Criterion, BIC = Bayesian Information Criterion, logLik = log likelihood [file peerj-10-13787-s007.pdf]

Table S3.

	Df	AIC	BIC	logLik	Deviation	Chi square	Df	P (>Chi square)
**A. Leaves**								
Null model	5	3454.6	3473.7	-1722.3	3444.6			
Time	8	3456.8	3487.4	-1720.4	3440.8	3.8	3	0.279
Null model	4	3500.1	3515.4	-1746.0	3492.1			
Site	8	3456.8	3487.4	-1720.4	3440.8	51.3	4	< 0.001
Null model	3	3498.5	3509.9	-1746.2	3492.5			
Site : Time	8	3456.8	3487.4	-1720.4	3440.8	51.7	5	< 0.001
**B. Branches**								
Null model	5	2667.4	2686.6	-1328.7	2657.4			
Time	8	2670.7	2701.3	-1327.3	2654.7	2.7	3	0.435
Null model	4	2666.2	2681.5	-1329.1	2658.2			
Site	8	2670.7	2701.3	-1327.3	2654.7	3.5	4	0.480
Null model	3	2664.8	2676.2	-1329.4	2658.8			
Site : Time	8	2670.7	2701.3	-1327.3	2654.7	4.1	5	0.538
**C. Reproductive**								
Null model	5	3171.4	3190.5	-1580.7	3161.4			
Time	8	3172.5	3203.1	-1578.2	3156.5	4.9	3	0.181
Null model	4	3184.3	3199.6	-1588.2	3176.3			
Site	8	3172.5	3203.1	-1578.2	3156.5	19.8	4	< 0.001
Null model	3	3183.5	3195.0	-1588.8	3177.5			
Site : Time	8	3172.5	3203.1	-1578.2	3156.5	21.0	5	0.091
**D. Miscellaneous**								
Null model	5	3225.8	3245.0	-1607.9	3215.8			
Time	8	3220.1	3250.7	-1602.0	3204.1	11.7	3	0.183
Null model	4	3239.8	3255.0	-1615.9	3231.8			
Site	8	3220.1	3250.7	-1602.0	3204.1	27.7	4	< 0.001
Null model	3	3240.6	3252.1	-1617.3	3234.6			
Site : Time	8	3220.1	3250.7	-1602.0	3204.1	30.5	5	0.087
