# Supplementary material for: Litter inputs and standing stocks in riparian zones and streams under secondary forest and managed and abandoned cocoa agroforestry systems
Source: PeerJ. 2022 Dec 1;10:e13787. doi: 10.7717/peerj.13787 (PMC9744167; doi:10.7717/peerj.13787)
Supplement: Supplemental Information 8 — AIC = Akaike Information Criterion, BIC = Bayesian Information Criterion, logLik = log likelihood [file peerj-10-13787-s008.pdf]

Table S4.

	Df	AIC	BIC	logLik	Deviation	Chi square	Df	P (>Chi square)
**A. Leaves**								
Null model	5	2055.4	2071.1	-1022.7	2045.4			
Time	8	2049.4	2074.5	-1016.7	2033.4	12.0	3	0.007
Null model	4	2086.4	2098.9	-1039.2	2078.4			
Site	8	2049.4	2074.5	-1016.7	2033.4	45.0	4	< 0.001
Null model	3	2088.9	2098.3	-1041.5	2082.9			
Site : Time	8	2049.4	2074.5	-1016.7	2033.4	49.5	5	< 0.001
**B. Branches**								
Null model	5	1905.2	1920.9	-947.57	1895.2			
Time	8	1910.4	1935.6	-947.22	1894.4	0.7	3	0.870
Null model	4	1937.9	1950.5	-964.95	1929.9			
Site	8	1910.4	1935.6	-947.22	1894.4	35.5	4	< 0.001
Null model	3	1936.1	1945.5	-965.06	1930.1			
Site : Time	8	1910.4	1935.6	-947.22	1894.4	35.7	5	0.099
**C. Reproductive**								
Null model	5	1653.2	1668.9	-821.62	1643.2			
Time	8	1657.1	1682.2	-820.55	1641.1	2.1	3	0.544
Null model	4	1671.3	1683.9	-831.66	1663.3			
Site	8	1657.1	1682.2	-820.55	1641.1	22.2	4	< 0.001
Null model	3	1670.0	1679.4	-832.00	1664.0			
Site : Time	8	1657.1	1682.2	-820.55	1641.1	22.9	5	< 0.001
**D. Miscellaneous**								
Null model	5	2200.7	2216.4	-1095.3	2190.7			
Time	8	2203.7	2228.8	-1093.8	2187.7	3.0	3	0.3905
Null model	4	2232.1	2244.7	-1112.1	2224.1			
Site	8	2203.7	2228.8	-1093.8	2187.7	36.4	4	< 0.001
Null model	3	2230.1	2239.6	-1112.1	2224.1			
Site : Time	8	2203.7	2228.8	-1093.8	2187.7	36.4	5	< 0.001
