# Supplementary material for: Litter inputs and standing stocks in riparian zones and streams under secondary forest and managed and abandoned cocoa agroforestry systems
Source: PeerJ. 2022 Dec 1;10:e13787. doi: 10.7717/peerj.13787 (PMC9744167; doi:10.7717/peerj.13787)
Supplement: Supplemental Information 9 — AIC = Akaike Information Criterion, BIC = Bayesian Information Criterion, logLik = log likelihood [file peerj-10-13787-s009.docx]

Table S5.

|  | Df | AIC | BIC | logLik | Deviation | Chi square | Df | P (>Chi square) |
| --- | --- | --- | --- | --- | --- | --- | --- | --- |
| **Terrestrial input** | | |  |  |  |  |  |  |
| null model | 5 | -31.9 | -12.8 | 20.9 | -41.9 |  |  |  |
| Time | 8 | -57.4 | -26.8 | 36.7 | -73.4 | 31.5 | 3 | < 0.001 |
| null model | 4 | -51.9 | -36.6 | 29.9 | -59.9 |  |  |  |
| Site | 8 | -57.4 | -26.8 | 36.7 | -73.4 | 13.5 | 4 | 0.009 |
| null model | 3 | -27.5 | -16.1 | 16.8 | -33.5 |  |  |  |
| Site : Time | 8 | -57.4 | -26.8 | 36.7 | -73.4 | 39.9 | 5 | < 0.001 |
| **Vertical input** | | |  |  |  |  |  |  |
| null model | 5 | 3123.5 | 3146.6 | -1556.8 | 3113.5 |  |  |  |
| Time | 8 | 3058.1 | 3095.0 | -1521.0 | 3042.1 | 71.5 | 3 | < 0.001 |
| null model | 4 | 3126.7 | 3145.2 | -1559.4 | 3118.7 |  |  |  |
| Site | 8 | 3058.1 | 3095.0 | -1521.0 | 3042.1 | 76.7 | 4 | < 0.001 |
| null model | 3 | 3164.3 | 3178.1 | -1579.1 | 3158.3 |  |  |  |
| Site : Time | 8 | 3058.1 | 3095.0 | -1521.0 | 3042.1 | 116.2 | 5 | < 0.001 |
| **Lateral input** | |  |  |  |  |  |  |  |
| null model | 5 | 502.9 | 522.0 | -246.5 | 492.9 |  |  |  |
| Time | 8 | 508.1 | 538.7 | -246.1 | 492.1 | 0.8 | 3 | 0.854 |
| null model | 4 | 544.7 | 560.0 | -268.4 | 536.7 |  |  |  |
| Site | 8 | 508.1 | 538.7 | -246.1 | 492.1 | 44.6 | 4 | < 0.001 |
| null model | 3 | 543.0 | 554.5 | -268.5 | 537.0 |  |  |  |
| Site : Time | 8 | 508.1 | 538.7 | -246.1 | 492.1 | 44.9 | 5 | < 0.001 |
| **Standing stock** | | |  |  |  |  |  |  |
| null model | 5 | 1415.6 | 1431.3 | -702.8 | 1405.6 |  |  |  |
| Time | 8 | 1419.3 | 1444.5 | -701.7 | 1403.3 | 2.3 | 3 | 0.050 |
| null model | 4 | 1454.4 | 1466.9 | -723.2 | 1446.4 |  |  |  |
| Site | 8 | 1419.3 | 1444.5 | -701.7 | 1403.3 | 43.0 | 4 | < 0.001 |
| null model | 3 | 1453.5 | 1462.9 | -723.7 | 1447.5 |  |  |  |
| Site : Time | 8 | 1419.3 | 1444.5 | -701.7 | 1403.3 | 44.1 | 5 | < 0.001 |
